# Combination of Sorafenib, Camrelizumab, Transcatheter Arterial Chemoembolization, and Stereotactic Body Radiation Therapy as a Novel Downstaging Strategy in Advanced Hepatocellular Carcinoma With Portal Vein Tumor Thrombus: A Case Series Study

**DOI:** 10.3389/fonc.2021.650394

**Published:** 2021-08-02

**Authors:** Yun Huang, Zeyu Zhang, Weijun Liao, Kuan Hu, Zhiming Wang

**Affiliations:** Department of Hepatobiliary Surgery, Xiangya Hospital, Central South University, Changsha, China

**Keywords:** camrelizumab, downstaging strategy, hepatocellular carcinoma, sorafenib, stereotactic body radiation therapy, transcatheter arterial chemoembolization

## Abstract

**Background and Aim:**

Although the treatment effect and availability of therapeutic options for advanced hepatocellular carcinoma (HCC) are limited, the downstaging strategy may improve patient prognosis. This study aimed to investigate the potential of combination therapy as a downstaging strategy for treating advanced HCC with portal vein tumor thrombus (PVTT).

**Methods:**

This retrospective case series included patients having advanced HCC with PVTT, who received the combination therapy of sorafenib, camrelizumab, transcatheter arterial chemoembolization (TACE), and stereotactic body radiation therapy (SBRT) from January 2019 to December 2019 in Xiangya Hospital, Central South University. The downstaging rate, treatment responses, progression-free survival (PFS), overall survival (OS), disease control rate, and toxicities were evaluated.

**Results:**

Of the 13 patients, HCC downstaging was achieved in 4 (33.3%) patients who later received hepatectomy. The overall response rate was 41.7%, and the disease control rate was 50.0%. The median PFS time was 15.7 months, with a 1-year PFS rate of 58.3%, whereas the median OS was not reached after 1 year (1-year OS, 83.3%). No severe adverse events or grade 3–4 adverse effect was observed in 12 of the 13 enrolled patients; therapy had to be discontinued in only one patient due to adverse events, who was excluded from the study. The most common adverse effect was fever (*n* = 4, 33.3%), followed by skin reaction (*n* = 3, 25%).

**Conclusion:**

A combination therapy comprising sorafenib, camrelizumab, TACE, and SBRT is an effective downstaging strategy for advanced HCC with PVTT and is associated with few adverse events.

## Introduction

Hepatocellular carcinoma (HCC) is the most common liver malignancy and the fourth leading cause of death worldwide (1). The early- and intermediate-stage HCC can be treated with hepatectomy and liver transplantation. However, the therapeutic options for advanced HCC are limited and provide inadequate treatment effect, resulting in poor patient prognosis.

Portal vein tumor thrombosis (PVTT) occurs in approximately 35%–50% of patients with HCC. It is a negative prognostic factor due to the increased risk of hematogenous tumor spread, leading to a high recurrence risk (2). Therapies such as transcatheter arterial chemoembolization (TACE), chemotherapy, radiotherapy, tyrosine kinase inhibitor (TKI), and immune checkpoint inhibitors (ICI) can be potentially used to treat advanced HCC. Studies on combination therapies such as ICI plus TKI and ICI plus local treatments have exhibited encouraging results to treat advanced HCC (3–5). More potential combinations are already under investigation.

Downstaging therapy, which can be achieved through neoadjuvant therapy, entails the application of certain treatments before definitive surgery to alleviate tumor burden and improve the outcome of the surgery (6). The treatment effect of surgery in patients with resectable advanced HCC is controversial, and the present guidelines do not recommend it because of a high recurrence rate (7). Thus, downstaging therapy may be a viable option for improving patient prognosis. However, effective downstaging strategies are still being explored.

Radiotherapy, TACE, and TKI in combination with ICI are increasingly used in advanced HCC. Sorafenib combined with TACE appeared to be a successful downstaging therapy for advanced HCC (8). Studies reported radiotherapy as an effective downstaging strategy in advanced HCC (9, 10). TKI in combination with ICI was also proposed as a potentially advantageous preoperative treatment (11).

Thus, the present study retrospectively investigated the role of a combination therapy of TKI, ICI, TACE, and radiotherapy as a downstaging strategy for treating advanced HCC with PVTT.

## Methods

### Patients

The present retrospective case series included patients having advanced HCC with PVTT, who received a combination therapy of sorafenib, camrelizumab, TACE, and stereotactic body radiation therapy (SBRT) in Xiangya Hospital, Central South University between January 2019 and December 2019. HCC was diagnosed using the noninvasive criteria of the European Association for the Study of Liver (EASL) guidelines (12). The tumor stage of HCC was determined using the Barcelona Clinic Liver Cancer (BCLC) system, whereas PVTT was assessed using the Cheng’s PVTT classification (13, 14). Patients with recurrence of HCC; history of anti-tumor therapy, including surgical tumor resection and systematic treatment; distant metastasis; Child-Pugh grade C; or hepatitis C virus or human immunodeficiency virus infection were excluded from the study. The ethics committee of the Xiangya Hospital of Central South University approved this study. Patient consent was waived due to the retrospective nature of this study.

### Study Design

Sorafenib (Nexavar, 200 mg, Bayer and Onyx) was administered orally as a TKI at a dose of 400 mg twice per day. The dose was reduced to 200 mg twice per day in the case of intolerable drug-related adverse events. Camrelizumab (Jiangsu Hengrui Medicine Co., Jiangsu, China), as an ICI, was administered intravenously at a dose of 200 mg for every 3 weeks. TACE was performed by supra-selective cannulation of the artery supplying the tumor with an injection of lipiodol with cisplatin. Radiotherapy was performed through SBRT using CyberKnife (Accuray, USA) with a total of 36–42 Gy in 4–5 fractions. Sorafenib and camrelizumab were initialized simultaneously, followed by TACE within 2 weeks and SBRT within 1 month. Sorafenib and camrelizumab were administered continuously until the occurrence of a serious adverse event, tumor progression, or a second surgery performed after successful downstaging.

### Clinical Data Collection and Definition of the Outcomes

Patients were continuously followed up every month after the initiation of therapy. Blood tests, including those for liver function and tumor biomarkers, and computed tomography (CT) were performed every 3 months, and MRI was performed as appropriate. The tumor response, rate of downstaging, progression-free survival (PFS), overall survival (OS), disease control rate (DCR), and adverse events were assessed. PFS was calculated from the first day of combination therapy administration to tumor progression or death of the patient, whereas the OS was calculated from the first day of combination therapy administration to death of the patient. DCR was defined as the percentage of patients who achieved complete response, partial response, and stable disease. The final follow-up was performed on October 29, 2020. Tumor response was determined using the modified response evaluation criteria in solid tumors (mRECIST), and adverse events were defined using the National Cancer Institute–Common Terminology Criteria for Adverse Events (NCI-CTCAE) version 5.0. Successful downstaging was defined as the disappearance of any intratumoral arterial enhancement of PVTT according to the mRECIST and EASL criteria (15, 16).

### Statistical Analysis

SPSS 23.0 (IBM, IL, USA) for Windows and Prism software (GraphPad Prism Software, CA, USA) were used to analyze the data and visualize the results. Quantitative data were expressed as the means ± standard deviation (SD), and categorical data were expressed as a number (percentage). Kaplan–Meier curves were used to illustrate the PFS and OS.

## Results

### Patient Characteristics

A total of 13 patients were enrolled in this study. One patient withdrew from the study due to intolerable skin reactions and was subsequently lost to follow-up; thus, the data of 12 patients were analyzed for this study (**Table 1**). Ten of the 12 patients were male (83.3%) with a mean age of 54.50 ± 9.91 years. All 12 patients had previously hepatitis B infection and 10 (83.3%) were diagnosed with liver cirrhosis. The mean tumor size was 8.66 ± 2.80 cm, and seven patients (58.3%) presented with multiple tumors. According to Cheng’s PVTT classification, two, six, and four patients were classified as Types I, II, and III, respectively. The mean levels of alanine aminotransferase, aspartate aminotransferase, and albumin were 68.22 ± 53.82 U/L, 64.10 ± 35.76 U/L, and 40.47 ± 5.45 g/L, respectively. Furthermore, the liver function of most patients (91.7%) was normal (Child-Pugh A class).

**Table 1:** Clinicopathological variables of enrolled patients with HCC.

Variable	Value (*n* = 12)
Age (year)	54.50 ± 9.91
Sex	
Male	10 (83.3)
Female	4 (16.7)
Ethnicity	
Asian	12 (100.0)
BMI	18.9 ± 3.0
HBsAg	
Negative	0 (0.0)
Positive	12 (100.0)
Liver cirrhosis	
No	2 (16.7)
Yes	10 (83.3)
Tumor size (cm)	8.66 ± 2.80
Number of tumors	
Single	5 (41.7)
Multiple	7 (58.3)
Cheng’s PVTT classification	
I	2 (16.7)
II	6 (50.0)
III	4 (33.3)
AFP (ng/mL)	
≤400	4 (33.3)
>400	8 (66.7)
ALT (U/L)	68.22 ± 53.82
AST (U/L)	64.10 ± 35.76
Albumin (g/L)	40.47 ± 5.45
Child-Pugh classification	
A	11 (91.7)
B	1 (8.3)

Data are expressed as mean ± standard deviation or n (%). AFP, Alpha-fetoprotein; ALT, Alanine aminotransferase; AST, Aspartate aminotransferase; BMI, body mass index; HBsAg, hepatitis B surface antigen; HCC, hepatocellular carcinoma; PVTT, portal vein tumor thrombus.

### Effectiveness of Combination Therapy

**Figure 1:**
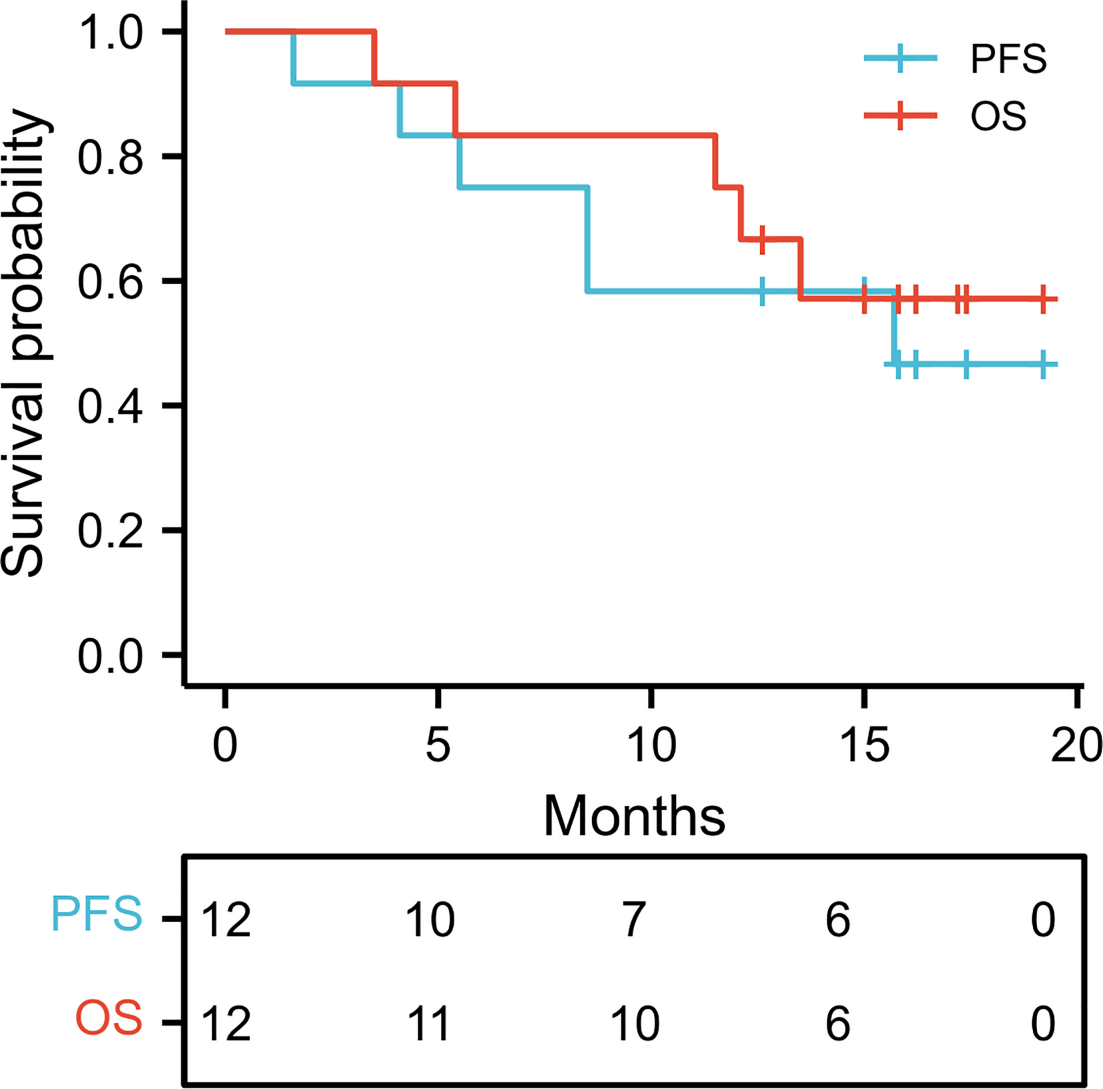
Kaplan–Meier curves of PFS and OS. OS, Overall survival; PFS, progression-free survival.

**Table 2** presents the effects of the treatment on the patients. Downstaging of HCC was achieved in four (33.3%) patients, who then received hepatectomy (three right and one left hemihepatectomy) without postoperative complications. Partial response and stable disease were achieved in one patient, respectively. Progressive disease was observed in six patients, of which five died by the last follow-up. The overall response rate (ORR) was 41.7%, whereas DCR was 50.0%. The median PFS time was 15.7 months, with a 1-year PFS rate of 58.3%. The median OS time was not reached with a 1-year OS rate of 83.3% (**Figure 1**). Among the four downstaged patients, the mean time to achieve downstaging was 5.36 ± 0.91 months. PVTT inactivation was confirmed by the subsequent pathological examination. Of the 12 patients, downstaging was achieved in 1 patient with PD after 6.5 months of therapy. However, he refused to undergo surgery, and tumor progression was detected in this patient after 2 months of continuing combination therapy. The pathological information of four downstaged patients is shown in **Table 3**.

**Table 2:** Efficacy of combination therapy in advanced HCC with PVTT.

Variable	Value (*n* = 12)
Tumor response	
Downstaged to surgery	4 (33.3)
Right hemihepatectomy	3 (25.0)
Left hemihepatectomy	1 (8.3)
Partial response	1 (8.3)
Stable disease	1 (8.3)
Progressive disease	6 (50.0)
Objective response rate (%)	41.7
Disease control rate (%)	50.0
Progression-free survival	
Median survival time [95% CI] (month)	15.7 [NR–NR]
Mean survival time [95% CI] (month)	13.1 [9.3–16.9]
1 year (%)	58.3
Overall survival	
Median survival time [95% CI] (month)	NR
Mean survival time [95% CI] (month)	15.0 [11.8–18.1]
1 year (%)	83.3

Data are expressed as n (%), %, or months (95% CI). HCC, Hepatocellular carcinoma; NR, not reached; PVTT, portal vein tumor thrombus.

**Table 3:** Pathological information of four patients downstaged to surgery.

Variable	Patient 1	Patient 2	Patient 3	Patient 4
Pathological diagnosis	HCC	HCC	HCC	HCC
Degree of differentiation	Low	Moderate	Low	High
Immunohistochemistry				
GPC-3	+	+	+	–
AFP	+	+	–	+
KI67	65%	10%	60%	2%
HepPar-1	+	+	+	+
Arg-1	+	+	+	+
CK7	–	–	–	–
CK19	–	–	–	–

HCC, Hepatocellular carcinoma.

### Adverse Events of Combination Therapy

No severe adverse events or grade 3 or 4 adverse effect was observed in the 12 evaluated patients. As mentioned previously, the therapy had to be discontinued only in one patient due to intolerable skin reactions. The most common adverse effect was fever (4 of 12, 33.3%), followed by skin reactions (3 of 12, 25%). Fatigue was observed in two patients, and vomiting, hypertension, diarrhea, and thrombocytopenia occurred in one patient each. Seven out of 12 evaluated patients were intolerant to the full dose of sorafenib, and the dose of sorafenib was reduced to 200 mg twice per day.

### Presentation of Two Downstaged Patients

**Figure 2:**
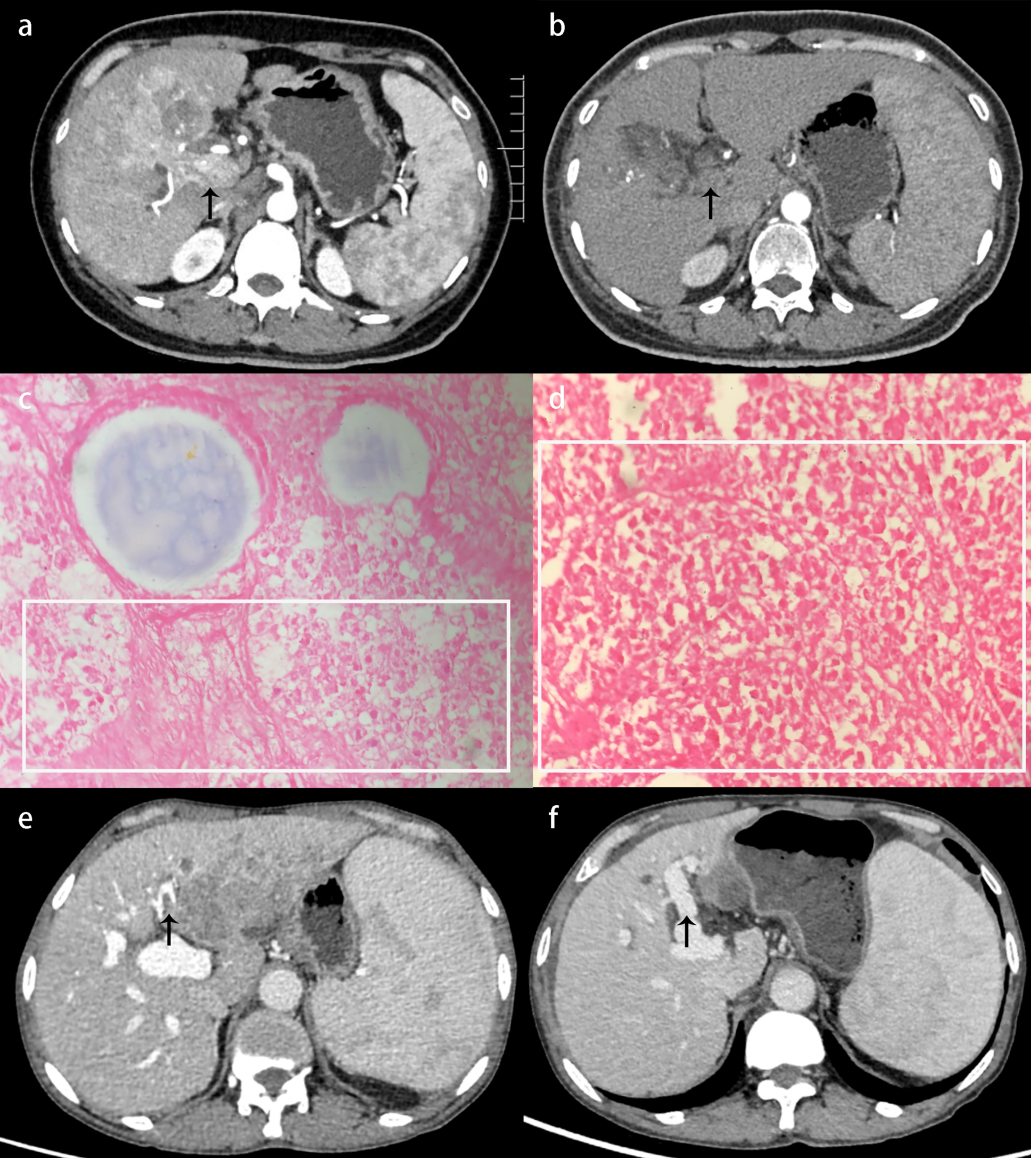
Presentation of two downstaged patients with HCC. **(A)** Before administering the combination therapy, contrast-enhanced CT exhibited tumor thrombus in the main trunk of the portal vein with enhancement during the arterial phase (black arrow). **(B)** After 4.5 months of the combination therapy, contrast-enhanced CT exhibited shrunken tumor size and necrotic tumor tissues in the portal vein without enhancement (black arrow). **(C)** Pathological examination confirmed HCC with massive necrosis (×400); the necrotic region is shown in a white rectangle. **(D)** Pathological examination confirmed that the PVTT was completely necrotic (×400); the necrotic region is shown in a white rectangle. **(E)** Contrast-enhanced CT showed tumor thrombus in the left main trunk of the portal vein with enhancement during the portal venous phase (black arrow). **(F)** After 6.5 months of the combination therapy, contrast-enhanced CT showed shrunken tumor size and absence of PVTT (black arrow). CT, Computed tomography; HCC, hepatocellular carcinoma; PVTT, portal vein tumor thrombus.

A 47-year-old female patient was admitted and diagnosed as having BCLC stage C HCC with PVTT (**Figure 2A**). After a comprehensive evaluation, the combination therapy was administered as described previously. After 4.5 months of therapy, the CT reexamination exhibited necrotic tumor tissues in the portal vein without enhancement (**Figure 2B**). A right hemihepatectomy was performed, and the subsequent pathological examination confirmed the diagnosis of HCC and complete necrosis of PVTT (**Figures 2C, D**). The patient recovered well with no recurrence at the last follow-up examination 1 year after the surgery. Similar alleviation was also shown in a 61-year-old male patient who experienced a shrunk tumor size and, more importantly, an absence of PVTT in the left main trunk (**Figures 2E, F**).

## Discussion

The treatment options for advanced HCC with PVTT are limited, and effective therapies need to be explored. The present study investigated the treatment effect of a new combination therapy consisting of sorafenib, camrelizumab, TACE, and SBRT in advanced HCC with PVTT. Our preliminary findings indicated that this combination could work as an effective downstaging strategy, with a downstaging rate as one out of three and an acceptable adverse event profile. This was the first study focusing on the treatment effect of this combination therapy for treating advanced HCC. This downstaging strategy could provide a potential chance to patients having advanced HCC with PVTT for receiving curative surgery to achieve a better prognosis.

Anti-PD-1 therapy is a promising treatment for advanced HCC. However, studies reported that only 15% of patients benefited from therapy alone (17, 18). Thus, efforts have been made to increase the treatment effectiveness by combining anti-PD-1 therapy with TKI. The combination of both apatinib and camrelizumab and that of lenvatinib and pembrolizumab showed a higher response rate (30%) than monotherapy, with a median PFS time of 8.6 months (5, 19). Efforts were also made to combine anti-PD-1 therapy with more than one therapy. Chiang et al. recently combined SBRT, TACE, and nivolumab in treating unresectable HCC and reported promising results (20). In the present study, we reported a higher ORR of 41.7% and a longer median PFS time of 15.7 months.

The application of radical surgery in advanced HCC with PVTT is still controversial. Surgery is not recommended as the first-line therapy among such patients in current guidelines; however, some studies have recommended radical surgery for a better prognosis (21). Downstaging therapies decrease the tumor burden and improve prognosis after surgery. A research confirmed that the survival data of downstaged HCC, followed by a salvage surgery, were similar to those of initially resectable HCC (22). Downstaging therapies reported in the literature include transarterial chemoembolization, transarterial radioembolization, radiotherapy, hepatic arterial infusion chemotherapy, and systemic therapy (23). Tabone et al. used radioembolization to downstage unresectable HCC, and 5 of 24 (20.8%) patients were downstaged to surgery (24). Lee et al. applied hepatic arterial infusion chemotherapy as a downstaging strategy in patients with advanced HCC, and 12 of 103 (11.7%) patients were downstaged to surgery (25). A case report demonstrated a superior treatment effect and fewer adverse events of lenvatinib in combination with nivolumab, followed by extended right hepatectomy (11). The rate of successful downstaging in the present study was 33.3%, which was higher than that of other therapies, indicating that our downstaging strategy could be an effective treatment option for patients with advanced HCC with PVTT.

TKI in combination with ICI was widely used for treating multiple solid tumors. Besides the simple combination of two drugs, a synergistic efficacy of TKI and ICI existed. Targeted therapies can enhance cancer immunity, such as tumor antigenicity, T-cell trafficking, or T-cell infiltration into tumors, which provides a rationale for combining them with checkpoint inhibitors or other cancer immunotherapies (26). Meanwhile, TACE increases tumor hypoxia, leading to the upregulation of hypoxia-inducible factor-1α, which upregulates the expression of vascular endothelial growth factor and platelet-derived growth factor and increases tumor angiogenesis, suggesting the combination of TACE and targeted therapy (27). Moreover, the promising data support the synergistic effect of immunotherapy and radiation in treating various solid tumors (28). However, the administration of radiotherapy led to the upregulation of PD-L1 (29), antigen-presenting cells, and effector T cells, and increased overall T-cell infiltration into tumors (30). These findings might explain the efficacy of the combination therapy suggested by the present study.

The time from initialization of the combination therapy to the confirmation of downstaging was mostly within 6 months. Thus, practitioners should pay attention to the response of patients during this time. In the present study, downstaging was achieved in one patient; however, the patient exhibited tumor progression after refusing to receive surgery. Thus, we suggested that surgery was beneficial for patients who achieved downstaging because the duration of effectiveness of sorafenib and camrelizumab was unpredictable. Future studies should focus on predicting the duration of effectiveness of ICI and TKI.

The present study had certain limitations. First, this was a retrospective single-arm study with a small cohort and thus carried a limited level of evidence. Second, we did not compare the effect of the standard dose of sorafenib and camrelizumab with that of the reduced dose because of the small cohort and insufficient information. Third, the long-term outcomes of downstaged patients after surgery were not investigated adequately because of the small sample size. Further studies with a larger sample size and longer follow-up time may strengthen the findings of this study. Finally, this study failed to explore the potential mechanisms underlyihng the synergistic effects of this combination therapy due to the retrospective nature of the study, which was essential for explaining the rationale of this combination therapy. Future studies should focus on this significant issue.

## Conclusion

The present study preliminarily established a combination therapy consisting of sorafenib, camrelizumab, TACE, and SBRT as an effective downstaging strategy in advanced HCC with PVTT. This is encouraging and deserves validation by prospective studies with a control arm and a larger cohort.

## Data Availability Statement

The raw data supporting the conclusions of this article will be made available by the authors, without undue reservation.

## Ethics Statement

The studies involving human participants were reviewed and approved by the ethics committee of Xiangya Hospital of Central South University. The ethics committee waived the requirement of written informed consent for participation.

## Author Contributions

All authors made substantive intellectual contributions to this study to qualify as authors. YH conceived the design of the study. ZW modified the design of the study. ZZ, WL, and KH performed the study, collected the data, and contributed to the design of the study. ZZ and WL analyzed the data. ZZ drafted the *Result*, *Discussion*, and *Conclusion* sections. WL drafted the *Methods* section. ZZ, KH, ZW, and YH edited the manuscript. All authors have agreed to be accountable for all aspects of the work in ensuring that questions related to the accuracy or integrity of any part of the work are appropriately investigated and resolved. All authors contributed to the article and approved the submitted version.

## Conflict of Interest

The authors declare that the research was conducted in the absence of any commercial or financial relationships that could be construed as a potential conflict of interest.

## Publisher’s Note

All claims expressed in this article are solely those of the authors and do not necessarily represent those of their affiliated organizations, or those of the publisher, the editors and the reviewers. Any product that may be evaluated in this article, or claim that may be made by its manufacturer, is not guaranteed or endorsed by the publisher.

## Abbreviations

BCLC, the Barcelona Clinic Liver Cancer; CT, computed tomography; HCC, hepatocellular carcinoma; ICI, immune checkpoint inhibitors; mRECIST, the modified Response Evaluation Criteria in Solid Tumors; NCI-CTCAE, The National Cancer Institute–Common Terminology Criteria for Adverse Events; ORR, overall response rate; OS, overall survival; PFS, progression-free survival; PVTT, portal vein tumor thrombus; SBRT, stereotactic body radiation therapy; SD, standard deviation; TACE, transcatheter arterial chemoembolization; TKI, tyrosine kinase inhibitor.
